# Neuroanatomy and Neuropsychology of Pain

**DOI:** 10.7759/cureus.1754

**Published:** 2017-10-06

**Authors:** Shehzad Khalid, R. Shane Tubbs

**Affiliations:** 1 Department of Anatomical Sciences, St. George's University School of Medicine, Grenada, West Indies; 2 Neurosurgery, Seattle Science Foundation

**Keywords:** neuroanatomy, neuropsychology, orbitofrontal cortex, pain, reinforcement learning, nociception, motor control

## Abstract

We have reviewed here the neuroanatomical and neuropsychological literature of the human brain and have proposed the various pain mechanisms that we currently know of. Essentially when tissue is damaged, peripheral nociceptors are activated continuously and prostanoids are hence produced. Nonsteroidal anti-inflammatory drugs (NSAIDs) and medications aim to target these prostanoids to treat the inflammatory component of pain. Normal pain tends to have a protective response. It is important for the nervous system to learn and recognize this painful stimulus earlier and quicker with repeated exposure to avoid tissue damage. This neuronal plasticity and gain in sensitivity result in sensitization of the nervous system, both centrally and peripherally and help in earlier detection of the pain sensation. However, persistent pain can become pathologic and will eventually result in the loss of protection pain offers to the body. Pain-related fear has been implicated in the transition from acute to chronic low back pain and the persistence of disabling low back pain, making it a key target for physiotherapy intervention. The current understanding of pain-related fear is that it is a psychopathological problem where people who catastrophise about the meaning of pain become trapped in a vicious cycle of avoidance behaviour, pain and disability, as recognised in the fear-avoidance model. We looked at how pain is perceived, especially in low-back pain patients. It has been hypothesized that individuals with low-back pain (LBP) can change their motor behavior, which is fundamentally an adaptation mechanism aimed at minimizing the real or perceived risk of further pain.

## Introduction and background

Acute and chronic pain puts a significant clinical, economic, and social burden on humanity [1]. Pain is the most common reason for a physician visit [1]. The Institute of Medicine (IOM) states that more than a 100 million Americans suffer from chronic pain [2]. Lost work-time exceeds 50 million days and lost productivity is 61.2 billion dollars per year [3]. The total direct and indirect cost of persistent pain is placed at $560-$635 billion annually, which far exceeds the cost of any of the other six major diseases including cardiovascular ($309 billion), neoplasms ($243 billion), injury and poisoning ($205 billion), endocrine, nutritional, and metabolic ($127 billion), gastrointestinal ($112 billion), and pulmonary ($112 billion) as published by the National Institute of Health (NIH) statistics [4]. It is also the most common cause of disability [1]. The comorbidities associated with pain, add to the burden of patients and families. These include opioid overuse, misuse, dependence and addiction, depression, poor social relationships and financial hardship [1]. We will review the mechanisms that result in acute and chronic pain as an end product.

## Review

Neuroanatomy and the process of nociception

The process by which the unpleasant noxious stimulus from the periphery is transmitted through the spinal cord and to various areas of the central nervous system resulting in the physiological sensation of pain and associated negative emotional response and memory, ultimately results in the sensation of pain. The first step in processing pain is the conversion of a stimulus in the periphery at nociceptive sensory fibers into an action potential. If a stimulus is of sufficient intensity that it reaches the threshold for an action potential, a nerve impulse is generated [5]. This propagates along the primary afferent fiber to the central nervous system. If the stimulus intensity increases, then additional nerve fibers and areas of the nervous system are recruited further [5]. Primary afferent fibers typically transmit information from more than one pain receptor secondary to afferent fiber branching. A single primary afferent and all its associated receptors have been labeled a sensory unit, and the area it collects information is known as the receptive field [5]. The larger the receptive field and more the overlap between adjacent fields, the harder it is for the sensory system to precisely localize the point of pain on the body. The first order (primary) afferent is a pseudounipolar neuron, where a single process divides into both a peripheral and a central axon. The cell bodies of these afferents are located in the peripheral nervous system in the posterior root or cranial root ganglia. The peripheral axon travels to the skin, muscle, tendon or joint where it branches into terminal fibers. Each terminal fiber ends on or forms what is called a somatosensory receptor. The central axon travels to the central nervous system [6]. Peripheral somatosensory fibers can be divided into three large groups. The first among them are the A-α, A-β, or A-γ fibers, which are large rapidly conducting myelinated fibers [7]. These are involved in touch and proprioception but are not involved with noxious perception. The second type is the A-δ fiber that can be small and slowly conducting. Some of these A-δ fibers are particularly involved with pain sensation. They consist of two types. Some have a high threshold, responding only to intense mechanical stimulation, while others respond also to heat at both noxious and non-noxious temperatures. The third type is the C fiber. C fibers are small, very slowly conducting unmyelinated fibers, of which most are involved in pain perception. They are polymodal and respond to all kinds of noxious mechanical, thermal, and chemical irritant stimuli. They are most often felt to be involved in burning pain sensation. There are a number of receptor subtypes. Heat is often mediated by transient receptor potential cation channel subfamily V member 1-3 (TRPV1-3) and TREK1, mechanical pressure is mediated by the MDEL7 and TREK1 and acid or chemical stimulus is mediated by acid-sensing ion channel (ASIC) [7]. The sensation of pain, which is termed “nociception”, is principally mediated through numerous intracellular and extracellular molecular messengers. Nociceptors transmit information via glutamate, an excitatory neurotransmitter, when they are activated by their particular requisite stimulus. Inflammatory mediators are also secreted at the site of injury to stimulate further nociceptor activation by secreting chemicals such as neurotransmitters (i.e., serotonin), lipids (i.e., prostaglandins), peptides (i.e., bradykinin), and neurotrophins (i.e., nerve growth factor) [7]. The presence of these molecules excites nociceptors or lowers their activation threshold, resulting in transmission of afferent signals to the dorsal horn of the spinal cord. They also initiate neurogenic inflammation, which is the process by which active nociceptors release neurotransmitters (i.e., substance P) from the peripheral terminal. This, in turn, causes vasodilation, which results in leaking of proteins and fluids into the extracellular space near the terminal end of the nociceptor. This then stimulates immune cells, which also further contribute to the inflammatory site reaction. Because of these neurochemical changes, the A-δ and C fibers are eventually activated. These nociceptors respond when there is a stimulus that causes tissue damage. Some of the involved substances include globulin and protein kinase, which are released from damaged tissue, and can actively produce pain. Arachidonic acid is also released during tissue damage, which is then metabolized to prostaglandins that block potassium efflux from nociceptors and makes them more sensitive. Histamine is also released when tissue damage stimulates mast cells to release it, which subsequently excites nociceptors and causes pain. Similarly, nerve growth factor is triggered by tissue damage or inflammation, which then binds to tropomyosin receptor kinase A (TrkA) receptors at the surfaces of nociceptors, activating them [1]. Substance P and calcitonin gene-related peptide are released by inflammation or tissue damage. These excite nociceptors and cause vasodilation and edema. Similarly, serotonin, acetycholine and adenosine triphosphate are released with tissue damage and also excite nociceptors. With increased metabolism, the release of lactic acid also excites nociceptors [1].

**Figure 1:**
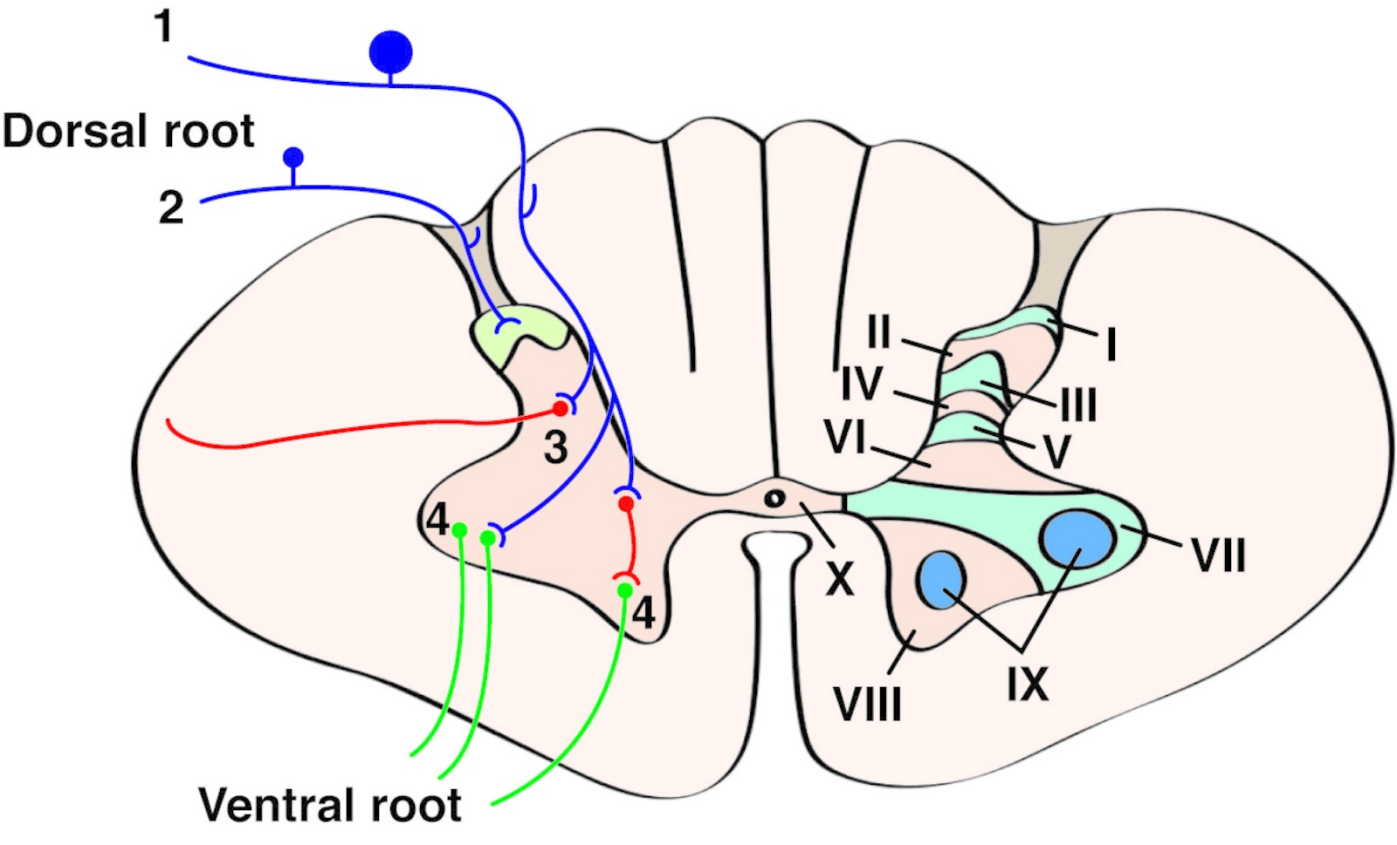
General cross-sectional anatomy of the spinal cord. Cells and connections in Laminae of Rexed are indicated. Large-diameter, heavily myelinated afferents (1) enter medially through the posterior funiculus, whereas small-diameter afferents (2) enter laterally near the substantia gelatinosa. This corresponds to the way tactile and proprioceptive information is processed, relative to pain and temperature information. These afferents then contact interneurons (3) and, in some cases, motor neurons (4) directly.

The primary afferent’s central process (cell body located in the dorsal root ganglion) joins a cranial or spinal nerve and enters the brainstem or spinal cord. There, it synapses with a secondary somatosensory neuron. Axons arriving in Rexed layers I and II (described later) release neurochemical agents such as glutamate, vasoactive peptide, somatostatin, calcitonin gene-related peptide, and substance P. This, in turn, activates nociceptive neurons in the spinal cord and can release glutamate, which further triggers the neurons and ultimately can make the N-methyl-D-aspartate (NMDA) receptors more sensitive to glutamate in a process called “central sensitization” [6]. However, before this sensitization, the information from activated nociceptor fibers is relayed to the spinal cord by the sensory cells located in the dorsal root ganglia. The lateral division of the dorsal root ganglion fibers contains most of the unmyelinated and small myelinated axons carrying pain and temperature information. These axons terminate in the Rexed laminae I, II and IV. The medial division of the dorsal root ganglion fibers carries information from primarily myelinated axons and terminates in the ipsilateral nucleus gracilis or nucleus cuneatus, though all fibers send collaterals to the different Rexed laminae [8]. The next step in the processing of pain signals occurs at the spinal cord. Its gray matter is formed in a pattern of lamination, where the cellular pattern of each lamina is composed of a different cytoarchitecture. Rexed proposed a classification based on the 10 laminae or layers that were related to a function of each lamina. Laminae I-IV are concerned with exteroceptive sensation and comprise the dorsal horn of the spinal cord. These are the main layers that process pain (Figure 1). Rexed lamina I cells respond mainly to noxious and thermal stimuli, and these axons join the contralateral spinothalamic tract. Lamina I corresponds to the nucleus posteromarginalis. Rexed lamina II corresponds to the substantia gelatinosa and responds to noxious stimuli. Axons in this layer receive information from sensory dorsal root ganglion cells and descending dorsolateral fasciculus fibers. They then send axons to Rexed laminae III and IV (this tract is called the fasciculus proprius). Lamina II has high concentrations of substance P and opiate receptors and is important in the modulation of sensory input. Therefore, this layer helps determine what pattern of sensations would be interpreted as painful. Laminae V and VI are mostly involved in proprioception. Rexed lamina V neurons receive information from A-β, A-δ and C axons carrying nociceptive information from visceral organs. Many cells from this layer project to the brainstem and the thalamus via the contralateral and ipsilateral spinothalamic tracts [1]. Lamina VII acts as a relay between muscle spindle to midbrain and cerebellum and can be considered an intermediate zone. In addition, all visceral motor neurons are located in lamina VII and innervate neurons in autonomic ganglia. Laminae VIII-IX encompasses the ventral horn of the spinal cord and contains α, β, and γ motor neurons whose axons innervate mostly striated or skeletal muscle. Lamina X surrounds the central canal and contains neuroglia [9].

**Figure 2:**
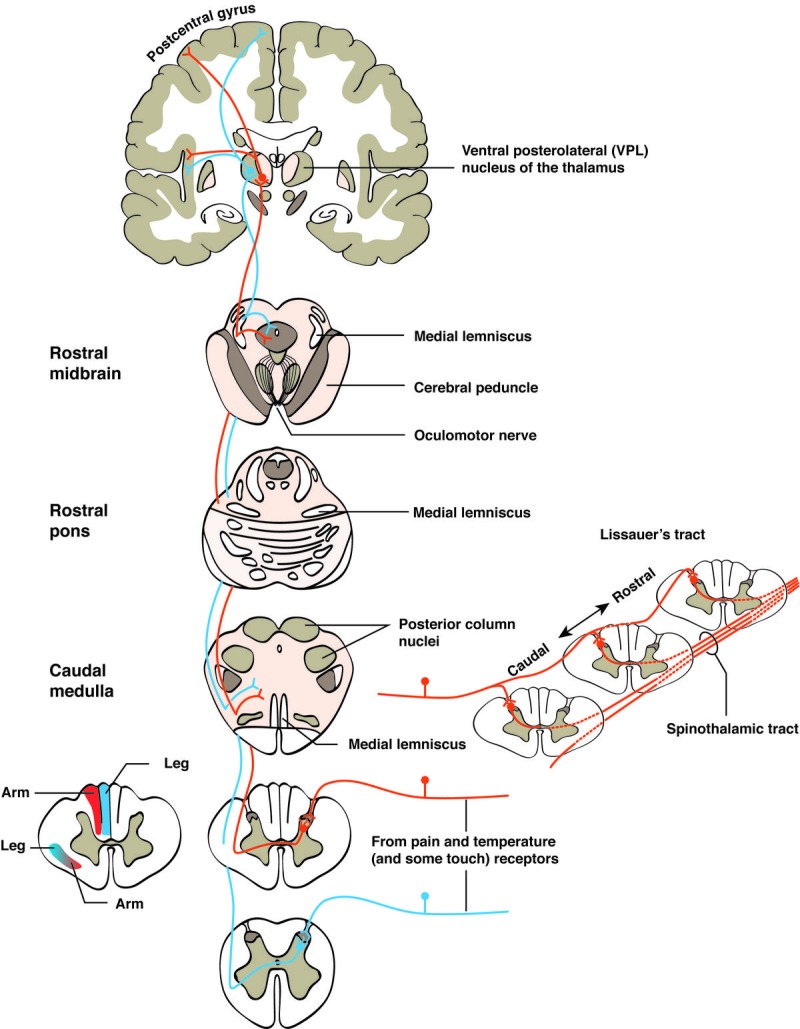
Spinothalamic tract. Pain, temperature, and some touch and pressure afferents end in the posterior horn. Second- or higher-order fibers cross the midline, form the spinothalamic tract, and ascend to the ventral posterolateral (VPL) nucleus of the thalamus (and also to other thalamic nuclei not shown). Thalamic cells then project to the somatosensory cortex of the postcentral gyrus, to the insula, and to other cortical areas (also not shown). Along their course through the brainstem, spinothalamic fibers give off many collaterals to the reticular formation (RF). The inset to the left shows the lamination of fibers in the posterior columns and the spinothalamic tract in a leg-lower trunk-upper trunk-arm sequence. The inset to the right shows the longitudinal formation of the spinothalamic tract. Primary afferents ascend several segments in Lissauerʼs tract before all their branches terminate; fibers crossing to join the spinothalamic tract do so with a rostral inclination. As a result, a cordotomy incision at any given level would spare most of the information entering the contralateral side of the spinal cord at that level, and to be effective, the incision must be made several segments rostral to the highest dermatomal level of pain.

Ultimately, there are ascending tracts that transmit sensory information from the periphery to the central nervous system. Fibers carrying information on two-point discrimination, tactile information, pressure, vibration and proprioception ascend through the dorsal column of the spinal cord. They form the gracile and cuneate fasciculi. Fibers carrying pain, temperature and crude touch information from both somatic and visceral structures ascend through the lateral spinothalamic tract. The anterior spinothalamic tract carries more pain, temperature and touch information in an ascending fashion to the brainstem and diencephalon (Figure 2) [9].

The spinothalamic tract afferents synapse in the posterior marginal nucleus of the posterior horn of the spinal cord. Their secondary afferent axons decussate in the spinal cord anterior white commissure and form the lateral part of the spinothalamic tract in the lateral funiculus. Therefore, fibers in the lateral spinothalamic tract are contralateral to their cells of origin and the body area they represent. These crossed second-order axons ascend in the spinal cord and brainstem as the spinothalamic tract. They terminate in the reticular formation of the brainstem or the periaqueductal gray of the midbrain or continue onto the diencephalon where they terminate in the ventral posterolateral nucleus of the thalamus or the intralaminar nucleus of the thalamus. Then axons from the thalamus (tertiary axons) travel in the posterior limb of the internal capsule and end in the postcentral gyrus and posterior paracentral lobule of the parietal lobe. These are the primary cortical areas receiving information about sharp pain and are organized in a somatotopic map to allow for accurate localization of pain. Intralaminar nuclei processes end in the insula and rostral cingulate gyrus, which are involved in receiving information about dull or deep-pain information. These areas are typically responsible for more poorly localized pain sensations that are longer lasting and associated with emotional features of pain [10]. Similarly, the spinal trigeminal path carries crude touch, pain and temperature information from the face. The primary spinal trigeminal afferents have A-δ and C peripheral axons that form free nerve endings in the dura mater and face. The primary afferents are located in the same nerves and ganglia as the typical sensory trigeminal pathway. Once they enter the brainstem, they form the spinal trigeminal tract, which is located from the mid-pons to C1 level of the spine. They then synapse on the spinal trigeminal nucleus. The secondary axons from the spinal trigeminal nucleus then decussate and form the ventral trigeminal lemniscus on the contralateral side to their origin. They then terminate in the brainstem reticular formation and then travel with afferents that leave the ventral trigeminal lemniscus and terminate near the periaqueductal gray or terminate in the ventroposteromedial (VPM) and the intralaminar nuclei of the thalamus. The VPM handles sharp, pricking gain, and the intralaminar nuclei process dull, burning, deep-aching pain, as well as temperature and crude touch. Then the tertiary afferent axons from the thalamus travel in the posterior limb of the internal capsule and end in multiple areas of the cerebral cortex. The VPM axons end in primary somatosensory cortex, which provides localization in the face for sharp and pricking pain. The intralaminar nuclei processes terminate in the cingulate gyrus and insula, which provide the sensory input for the perception of facial pain from the various tracts: dull, temperature, crude touch, etc. There are a number of receptor types involved in the above afferent pain processing signals. Signals transduced up the spinothalamic tract result in release of norepinephrine from the locus coerulus neurons projecting to the thalamus. This then relays nociceptive information to the somatosensory cortex, hypothalamus and hippocampus. Therefore, norepinephrine affects how nociceptive information is relayed for processing in cortical and subcortical brain regions [11].

However, there is a much more complicated interplay in the descending pain systems. Opioid receptors in the peripheral and central nervous systems result in inhibition of pain processing and analgesia when stimulated by opiates or endogenous opiates such as endorphin, encephalin or dynorphin, which are governed by the descending modulatory pain system. Pain modulation involves γ-aminobutyric acid (GABA) which further heightens the descending inhibition of spinal nociceptive neurons. Terminals of the descending pathway originate in the rostroventral medulla and other brainstem nuclei as well as the nucleus tractus solitarus and the parabrachial nucleus, the dorsal reticular nucleus, the hypothalamus, and the cortex [1]. These interact with the afferent fibers, interneurons, and projection neurons of the dorsal horn. Actions at these sites either suppress or enhance passage of nociceptive information to the periaqueductal gray, nucleus tractus solitarus, amygdala and other structures involved in secondary processing. They transfer nociceptive information to corticolimbic regions and interact with other areas to modulate the activity of the descending pathways [12].

**Figure 3:**
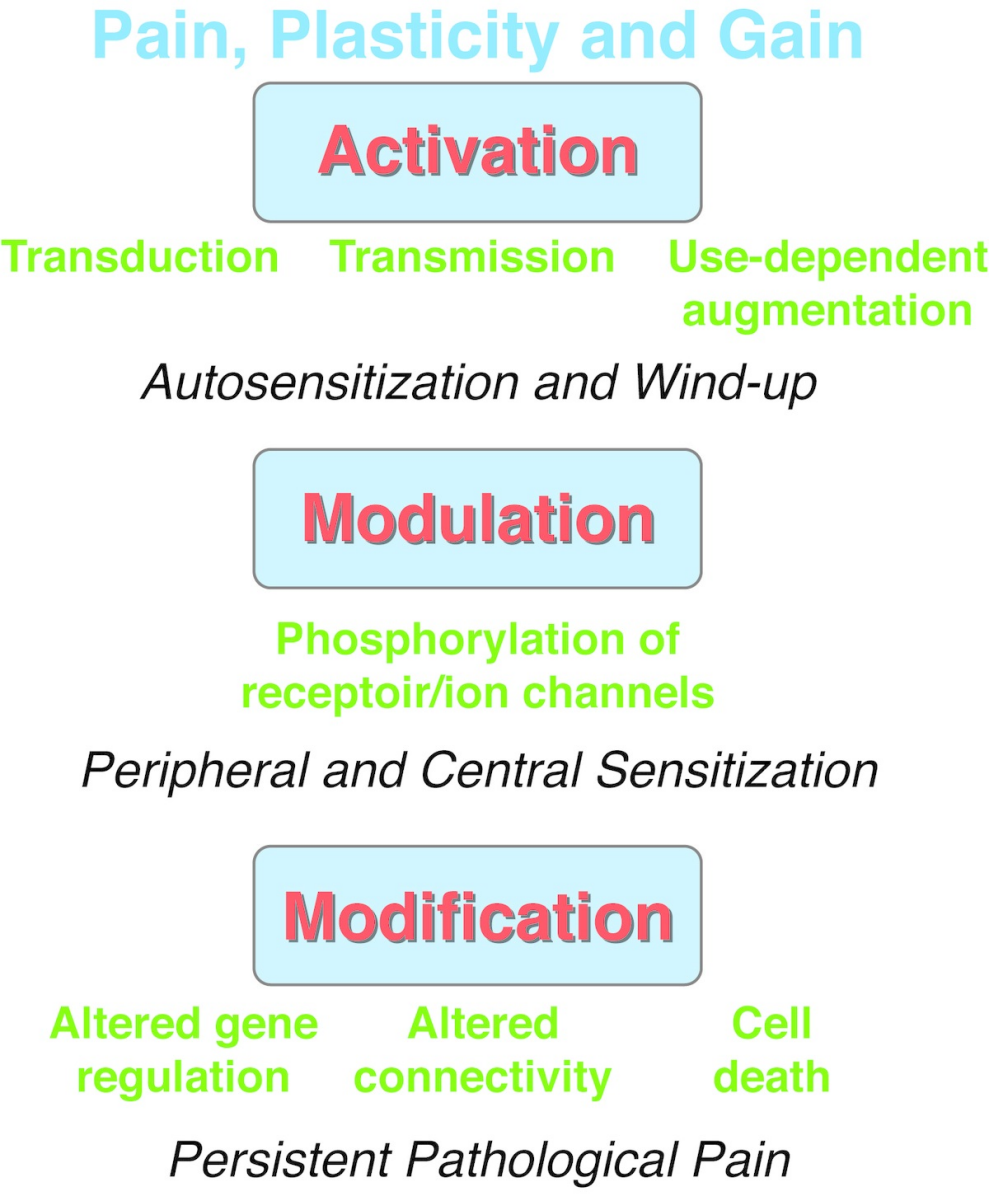
Pain, plasticity and gain. The three forms of neural plasticity that increase gain in the somatosensory system to produce pain hypersensitivity are illustrated, highlighting changes they produce and their effects on pain transmission.

As discussed previously, normal pain has a protective response. It is essential for the nervous system to learn and recognize this painful stimulus earlier and quicker with repeated exposure to avoid tissue damage. This neuronal plasticity and gain in sensitivity result in sensitization of the nervous system, both centrally and peripherally and help in earlier detection of the pain sensation. However, persistent pain could become pathologic and would result in the loss of the protection pain offers to the body. Persistent pain is a disease in itself unlike pain sensation, which is a symptom of the underlying disease condition. The following are the classically described stages of pain hypersensitivity progression from being a symptom to a disease. This activity-dependent neuroplasticity at the nociceptor is defined as “autosensitization”, where the threshold of the nociceptor transducers is reduced. If a stimulus activates the peripheral nervous system repeatedly without the activation of the transducers, it increases the sensitivity of the terminal membrane. This process is called “heterodesensitization” [1]. This is a process that sets in rapidly secondary to conformational changes in protein or calcium channels and is readily reversible. When this process happens at the dorsal ganglion level, this is called the “Windup” phenomenon [1]. Intense noxious stimulus results in the release of neuromodulators, glutamate, and activation of NMDA channels resulting in a temporal summation of the slow excitatory postsynaptic potential, longer neuronal depolarization, and resulting windup of the action potential (Figure 3) [13]. Clinically, activation produces the process of “allodynia”, which is pain hypersensitivity in the setting of a nonpainful stimulus, and “hyperalgesia”, which is an increased pain response in the setting of less-painful stimulus [13]. These are both reversible with removal of the pain stimulus. Peripheral sensitization is initiated by the intracellular contents such as adenosine, bradykinin, histamine, cytokines, prostaglandins, and growth factors stimulating the nociceptor terminal and transducers. With ongoing pain stimulus, the pain response is sustained secondary to phosphorylation of the receptor or ion channels or regulatory proteins in the primary sensory neurons. Simultaneous intracellular pathways through serine or threonine and tyrosine kinase cascades result in the more intense and sustained depolarization currents [13]. Central sensitization is the result of enhanced peripheral inputs over a sustained period of time to the dorsal horn neurons. This resultant intracellular pathway is similar to peripheral sensitization. Depressed “inhibition” of the central GABA or glycinergic pathways further activates the central sensitization process. This activation confined to a single synapse results in a “homosynaptic” transmission. When it spreads to adjacent neurons, it is called “heterosynaptic” transmission. This heterosynaptic transmission results in the spreading of pain in a nondermatomal fashion beyond the extent of the injury. NMDA channels activated by glutamate, when phosphorylated, result in increased channel activation. Subthreshold stimulus now can activate these dorsal neurons forming the basis for central sensitization process [13]. Chronic exposure to noxious stimulus resulting in inflammatory or neuropathic pain results in a more permanent structural and functional neuronal alteration. Changes are noticed in the synaptic neuromodulators, terminal membrane ion channels, G protein-coupled receptors and growth-associated proteins and structural proteins. Transcription changes in these neurons happen from the retrograde transport of these abnormal targets – derived growth factors and abnormal electrical activity from activation of voltage-gated calcium changes [14]. MAPK/pCREB cascade has been implicated in this process [15]. Both constitutively expressed genes and the induction of new genes cause the phenotypic shift of the neurons. Nerve injury also induces reorganization of the A fibers in the dorsal horn cells due to delayed loss of C fibers. This results in A fibers synapsing at the C fibers’ location in the spinal cord and expressing pain-related neuromodulators [16]. Ultimately this causes the persistence of intractable neuropathic pain.

Neuropsychology of pain-related fear and adaptations

Now that the mechanism of pain has been thoroughly explained, let’s take a look at how pain is instilled and creates fear. Pain-related fear is implicated in the transition from acute to chronic low-back pain and the persistence of disabling low back pain, making it a key target for physiotherapy intervention. The current understanding of pain-related fear is that it is a psychopathological problem where people who catastrophise about the meaning of pain become trapped in a vicious cycle of avoidance behaviour, pain and disability, as recognised in the fear-avoidance model. However, there is evidence that pain-related fear can also be seen as a common sense response to deal with low-back pain, for example, when one is told that their back is vulnerable, degenerating or damaged. In this instance avoidance is a common sense response to protect a ‘damaged’ back. While the fear-avoidance model proposes that when someone first develops low-back pain, the confrontation of normal activity in the absence of catastrophising leads to recovery, the pathway to recovery for individuals trapped in the fear-avoidance cycle is less clear. Understanding pain-related fear from a common sense perspective enables physiotherapists to offer individuals with low back pain and high fear a pathway to recovery by altering how they make sense of their pain. Drawing on a body of published work exploring the lived experience of pain-related fear in people with low-back pain, this clinical commentary illustrates how Leventhal's Common Sense Model may assist physiotherapists to understand the broader sense-making processes involved in the fear-avoidance cycle and how they can be altered to facilitate fear reduction by applying strategies established in the behavioural medicine literature [17]. A recent study done by Mohammadi, et al. investigated the mediating role of pain behaviours in the association between pain catastrophising and pain intensity and explored the moderating role of family caregivers’ responses to pain in the link between pain behaviours and pain intensity [18]. The sample consisted of 154 chronic pain patients and their family caregivers. Patients completed questionnaires regarding pain intensity, pain catastrophising, pain behaviours and their caregivers’ responses to their pain. Family caregivers reported their responses to the patients’ pain in return. The results showed that pain catastrophising was associated with pain intensity (r = 0.37) and pain behaviours partly mediated this association. The positive association between pain behaviours and pain intensity was significant only if patients reported that their family caregivers showed high levels of solicitious and distracting responses (effect = .58) and if caregivers reported to show high levels of solicitous responses (effect = .51) [18]. No support was found for negative responses as a moderator neither based on patients’ perception of negative responses nor based on caregivers’ perception of negative responses [18]. The findings were in line with the idea that family caregivers’ solicitous and distracting responses convey to patients that their condition is serious, which may reinforce patients’ pain and pain behaviours, especially in those who catastrophise [18].

Looking at low-back pain (LBP) specifically, you can see that it is still widely prevalent and globally, is the leading cause of years lived with disability due to functional limitations, limited benefits of treatment, and frequent recurrence [19]. Changes in motor behaviour in individuals with low-back pain are adaptations aimed at minimizing the real or perceived risk of further pain. For all intents and purposes, in reinforcement learning, a reward (positive reinforcement) or the absence or reduction of cost (negative reinforcement) increases the likelihood that a performed behaviour will be repeated and thus learned. In this context, movement-related pain may function as a negative reinforcement and the sense of being able to prevent pain provocation as a positive reinforcement. Motor control can be considered as the outcome of a learning process aimed at optimizing a combination of costs and rewards. Although movement patterns may differ between individuals, patients with LBP will tend to, however, control posture and movement more rigidly. Through reinforcement learning, pain and subsequent adaptations result in less dynamic motor behaviour, leading to increased loading and impoverished sensory feedback, which contributes to cortical reorganization and proprioceptive impairments that reduce the ability to control lumbar movement in a robust manner. Motor control in LBP is changed at many levels of the nervous system. Studies of individuals with and without LBP have reported differences in voluntary trunk muscle activation [20], trunk muscle reflexes [21], trunk kinematics [22], and in cortical mapping of sensory inputs and motor outputs to the trunk [23, 24]. However, the literature is far from consistent regarding the nature of these differences. According to a systematic review, there is support of both an increase and a decrease of trunk muscle activation in individuals with LBP [20]. It can be hypothesized that changes in motor control with LBP reflect functional adaptations acquired through reinforcement learning. These secondary, long-term effects may contribute to recurrence and chronicity of back pain. It has been suggested that movement planning occurs sequentially at two hierarchical levels: initially to plan the kinematic trajectory and subsequently to plan a muscle recruitment pattern that fits the planned kinematic trajectory [25]. In the present context, adaptation of motor control to changing conditions, in our case to the presence of nociception from the spine and the perception of LBP, is of particular interest. Many studies of reinforcement learning have addressed adaptation in the control of goal-directed arm movements to mechanical perturbations, to converge on a near straight-line hand trajectory that closely resembles the unperturbed trajectory [25]. Although nociceptive afference may lead to the perception of pain, the presence, intensity, and nature of this perception are shaped by cognitive factors, which include the expectation of pain [26]. The objective may be to minimize the perception of pain instead of the nociceptive input, or to maximize perceived safety rather than actual robustness of the motor strategy. This introduces the possibility for a feedback mechanism, where one change to the motor strategy leads to a perception of increased safety or decreased pain. This may explain why increasing muscle activity can reduce perceived pain under a constant nociceptive input [27]. A recent study applied a force field to the ankle during gait and provided noxious input to the tibialis anterior muscle. Participants with and without pain adapted quickly to the force field to continue walking without loss of balance, but only the participants without pain were able to continue to refine their adaptation; participants with pain continued to use their initially adopted solution. Both reduced variance of muscle recruitment patterns after administration of noxious stimulus to the low back [28] and increased variance have been reported [29]. Individuals with chronic LBP have been reported to display lower variability of muscle recruitment [30] and lower variability of trunk kinematics [31] than individuals without pain. However, opposite findings have also been reported [32]. The effects of LBP on trunk muscle activity [33] and trunk displacement after perturbations [34] are far more pronounced in participants with high scores on pain catastrophizing or fear of movement. Similarly, delayed deep muscle activation was more noticeable in participants with a high fear of pain [35] and variability was decreased in participants with negative pain-related perceptions [36], which persisted after the pain had resolved in these participants [37]. Further studies still need to be done to investigate various other factors in the processing of pain. Particularly, manipulation of nociception and pain as a function of motor behaviour could be used to shape objective functions and, when used as such, test what strategies are in place to avoid the painful stimulus developing through exploration and reinforcement.

Pain management

**Figure 4:**
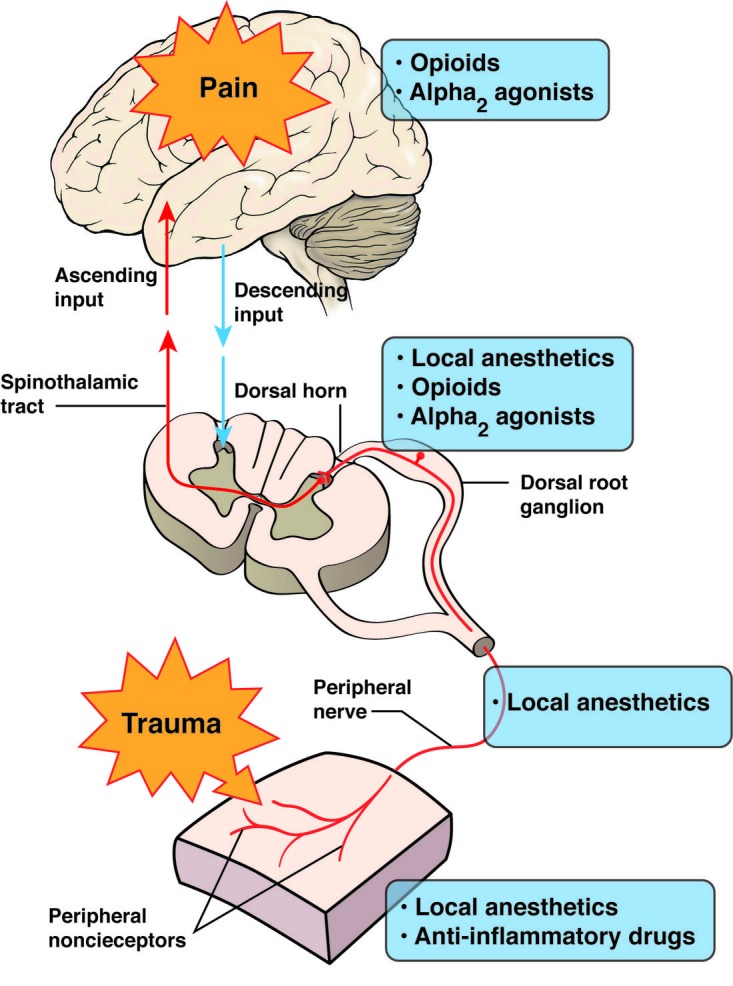
Target sites for management of pain. Pharmacologic approaches to management of pain are based on location in the nervous system.

As explained earlier in this article, there are a number of complicated, interconnected pathways through which the body perceives pain. There are a number of possible sites for intervention to reduce pain, including those that are peripherally mediated and those that are centrally mediated. Pharmacologic agents targeting various sites of the nervous system based on etiology have the best possible effect on treating both acute and chronic pain. However, a systems-based multidisciplinary approach, including pharmacology, rehabilitation, psychology pain coping skills and alternate and complementary therapies, would be the best approach for managing chronic persistent pain (Figure 4).

Medications that can block sensory hyperexcitability include anticonvulsants, antidepressants, and analgesics. They exert their effects on calcium channels, sodium channels, monoamine uptake mechanisms, and G-protein-coupled membrane receptors typically. Different types of pain may respond better to particular agents. For example, neuropathic pain may respond best to tricyclic antidepressants serotonin and norepinephrine reuptake inhibitors (SNRIs), or anticonvulsants. Osteoarthritis may respond best to acetaminophen, nonsteroidal anti-inflammatory drugs (NSAIDs), or tramadol. Fibromyalgia may respond best to muscle relaxants, tricyclic antidepressants, selective serotonin reuptake inhibitors, or serotonin and norepinephrine reuptake inhibitors in addition to tramadol or anticonvulsants. Low-back pain may respond best to NSAIDs, acetaminophen, muscle relaxants, or tramadol [38].

When tissue is damaged, peripheral nociceptors are activated essentially continuously. They produce chemicals in and around the damaged area that leads to the release of cytokines, prostanoids, and growth factors. The prostanoids are the target of NSAIDs and medications that aim to treat the inflammatory component of pain. In addition, abnormal firing of the sodium channels may result in pain (related to local tissue damage and inflammatory reactions) as well. Sodium channel blockers, such as lidocaine or carbamazepine, are used to block this aspect of nociception. Nerve trauma causes increased numbers of calcium channels to be expressed, which leads to release of more neurotransmitters such as glutamate and substance P. In the spinal cord, the release of peptides and glutamate causes activation of multiple receptors, but most notably the NMDA receptor. This releases glutamate and can therefore generate spinal hypersensitivity. Medications that block excitability may be effective, as are those that increase inhibition in the spinal cord. Ketamine works to modulate the NMDA-receptor-driven excitation. For example, opioids act via presynaptic and postsynaptic inhibitory effects on central and peripheral C-fiber terminals, spinal neurons, and supraspinal mechanisms targeting the descending pain modulatory system [11]. This is most notable in the periaqueductal gray, an area highly involved in processing placebo analgesia [39]. Gabapentin decreases excitatory input also by binding to calcium channels and disrupting trafficking of the channel to the synaptic membrane, thereby reducing neurotransmitter release. Antidepressants that inhibit reuptake of serotonin and noradrenaline (i.e., duloxetine) interact in a spinal cord-brain-spinal cord loop that includes central areas important in emotional and aversive responses to pain. Those central areas are activated by pain (which shifts the balance from noradrenergic inhibition toward serotonergic facilitation), but also by top-down processes such as fear and anxiety [38]. Nonpharmalogic interventions such as cognitive behavioral therapy and mindfulness techniques improve pain by working on these top-down processes also [39]. Still much remains to be discovered about the many functions of the brain in the processing of pain, and new evidence from functional neuroimaging and clinical neuropsychology is affording new insights of the human cortex [40].

## Conclusions

Pain is a protective response, which alerts the nervous system to potential tissue damage. Acute and chronic pain imposes a significant clinical, economic, and social burden on society. Chronic pain is the most common cause of disability. The direct and indirect costs far exceed another disease condition. The cascade of pain perception begins with a noxious stimulus either inflammatory or neuropathic. Multiple systems and pathways govern this pain perception and are both physiological and psychological in nature. However, persistence of stimulus results in neuroplasticity with increased neuronal sensitivity and resultant gain in action potentials inducing the persistence of pain. Further exposure results in neuronal phenotypic changes, which are initially reversible but with time are irreversible. Neuronal degeneration causes the remainder of the neurons to undergo structural reorganization. Cortical reorganization connects pain sensation to affect the memory. A proper understanding of the pain pathogenesis and neuroplasticity is essential to differentiate pain as a symptom of an underlying disease versus persistent pain as a disease in itself, because their treatment approaches are vastly different.
